# Cancer stem cells from human glioblastoma resemble but do not mimic original tumors after *in vitro* passaging in serum-free media

**DOI:** 10.18632/oncotarget.11676

**Published:** 2016-08-29

**Authors:** Noemí García-Romero, Carmen González-Tejedo, Josefa Carrión-Navarro, Susana Esteban-Rubio, Gorjana Rackov, Vanessa Rodríguez-Fanjul, Jorge Oliver-De La Cruz, Ricardo Prat-Acín, María Peris-Celda, David Blesa, Laura Ramírez-Jiménez, Pilar Sánchez-Gómez, Rosario Perona, Carmen Escobedo-Lucea, Cristobal Belda-Iniesta, Angel Ayuso-Sacido

**Affiliations:** ^1^ Instituto Madrileño de Estudios Avanzados, IMDEA Nanociencia, Madrid, Spain; ^2^ Centro Nacional de Biotecnología (CNB-CSIC), Madrid, Spain; ^3^ Fundación de Investigación HM Hospitales, HM Hospitales, Madrid, Spain; ^4^ Instituto de Medicina Molecular Aplicada (IMMA), School of Medicine, San Pablo-CEU University, Campus de Montepríncipe, Madrid, Spain; ^5^ International Clinical Research Center, Center for Translational Medicine, St. Anne's University Hospital, Brno, Czech Republic; ^6^ Neurosurgery Department, Hospital Universitario la Fe de Valencia, Valencia, Spain; ^7^ Genetic and Genomic Unit, Fundación Centro de Investigación Príncipe Felipe, Valencia, Spain; ^8^ Neuro-oncology Unit, Instituto de Salud Carlos III-UFIEC, Madrid, Spain; ^9^ Instituto de Investigaciones Biomédicas, CIBERER, CSIC/UAM, Madrid, Spain; ^10^ Division of Biopharmaceutics and Pharmacokinetics, University of Helsinki, Helsinki, Finland

**Keywords:** cancer stem cells, glioblastoma, genetic alterations, drug discovery, primary cell culture

## Abstract

Human gliomas harbour cancer stem cells (CSCs) that evolve along the course of the disease, forming highly heterogeneous subpopulations within the tumour mass. These cells possess self-renewal properties and appear to contribute to tumour initiation, metastasis and resistance to therapy. CSC cultures isolated from surgical samples are considered the best preclinical *in vitro* model for primary human gliomas. However, it is not yet well characterized to which extent their biological and functional properties change during *in vitro* passaging in the serum-free culture conditions. Here, we demonstrate that our CSC-enriched cultures harboured from one to several CSC clones from the human glioma sample. When xenotransplanted into mouse brain, these cells generated tumours that reproduced at least three different dissemination patterns found in original tumours. Along the passages in culture, CSCs displayed increased expression of stem cell markers, different ratios of chromosomal instability events, and a varied response to drug treatment. Our findings highlight the need for better characterization of CSC-enriched cultures in the context of their evolution *in vitro,* in order to uncover their full potential as preclinical models in the studies aimed at identifying molecular biomarkers and developing new therapeutic approaches of human gliomas.

## INTRODUCTION

Glioblastoma (GBM) is the most common and devastating brain tumor in human adults [1], with an incidence ranging from 0.59 to 3.69 per 100 000 persons/year [2]. The average survival does not exceed 15 months even after extensive surgery followed by radiotherapy alone or in combination with Temozolomide treatment [3–5], and just 0.05% to 4.7% of patients survive 5 years after diagnosis [6, 7].

High lethality of GBM might be partly attributed to a small population of tumour cells, termed Cancer Stem Cells (CSCs), which drive tumour initiation and maintenance [8–11]. Current approaches to treat GBM patients have little success, possibly due to the fact that the CSC subpopulation is refractory to both chemo- and radiotherapy. Growing evidence points to CSCs as a more reliable preclinical GBM model than traditional cancer cell lines; therefore, many efforts have been made to isolate and culture CSCs in order to study their contribution to the tumorigenic processes, as well as to identify new therapeutic targets and biomarkers for diagnostics, prognostics, GBM stratification, treatment selection, and follow-up response to therapy.

CSCs display intra- and inter-tumour heterogeneity [12, 13], carry genomic and genetic alterations found in the original tumour and phenocopy their critical histopathological features when grown in serum-free media [14]. Under these culture conditions, CSC gene expression patterns resemble the original tumour more closely than those of other established tumour cell lines [15, 16]. Comparative analysis of different CSC collections reveals at least two different CSC subtypes: the first one with a proneural-like phenotype, and a highly invasive behaviour, and the second one with a mesenchymal-like phenotype and a nodular pattern with minimal invasiveness [17–19]. In addition, mRNA expression profiling and cytogenetic analysis of 48 glioma surgical samples from The Cancer Genome Atlas Research Network (TCGA) [20, 21], suggests there are four different CSC subtypes: proneural, mesenchymal, classical and neural [22], thus emphasizing the value of CSCs as preclinical models for GBMs.

CSCs are dynamic systems susceptible to evolution in culture, resulting in molecular alterations not found in the original tumours [19]. Their response to functional assays, such as tumour cell migration and dissemination, proliferation or drug sensitivity, might thus experience important changes as they evolve, affecting the reproducibility of the results as well as their capacity to model the original tumours. Therefore, in order to define the best biologically and clinically relevant conditions for a given experiment, the study of GBM CSC phenotypic and molecular dynamic in culture is essential.

Here, we present a collection of results showing that serum-free media selected CSC-enriched cultures ranging from homogeneous to quite heterogeneous populations from the original tumour. As they evolved *in vitro*, these cells displayed increasing stemness marker expression, as well as different ratios of chromosomal instability events. They reproduced at least three different dissemination patterns found in the original tumours, but exhibited irregular response to drugs between passages. Our findings highlight the need to characterize a large number of CSC-enriched cultures, both in the context of their original tumours and their *in vitro* evolution, in order to take full advantage of these preclinical tumour models for developing new therapeutic approaches.

## RESULTS

### Isolation and characterization of CSC cultures from human surgical samples

**Figure 1:**
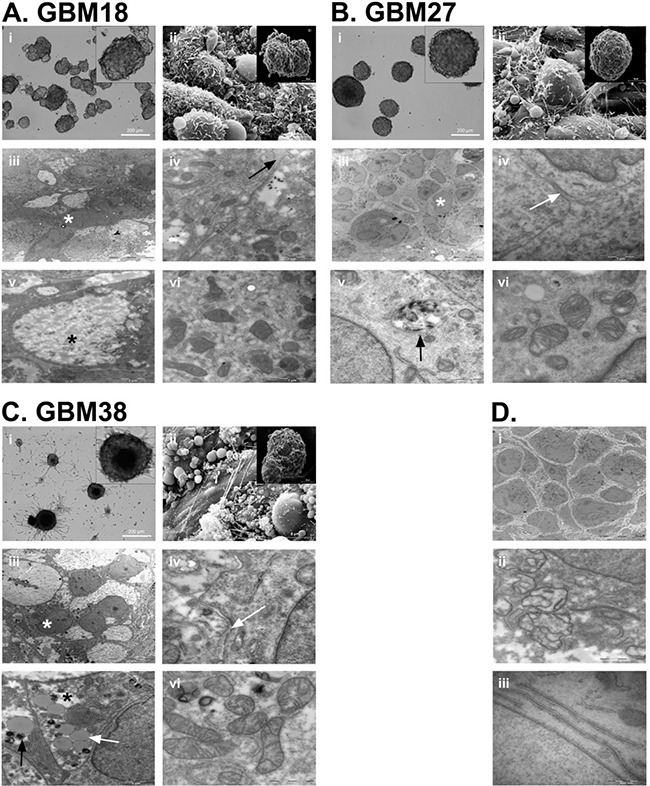
Neurosphere architecture and cell morphology analysis of GBM18, GBM27 and GBM38 CSC-enriched cultures **A.** GBM18 neurosphere morphology under the OM (*i*), SEM (*ii*) and TEM (*iii-vi*). Black arrow shows extracellular matrix (*iv*), white asterisk shows clear cytoplasm cells (*iii*) and black asterisk shows representative polysaccharide intracellular deposits (5). **B.** GBM27 neurosphere morphology under the OM (*i*), SEM (*ii*) and TEM (*iii-vi*). Black arrow shows extracellular matrix (*iv*), white asterisk shows clear cytoplasm cells (*iii*) and black asterisk shows representative electro-dense intracellular deposits (*v*). **C.** GBM38 neurosphere morphology under the OM (*i*), SEM (*ii*) and TEM (*iii-vi*). White arrow shows extracellular matrix (*iv*) and representative lipid drops (*v*). Black arrow shows representative electro-dense intracellular deposits (*v*). White asterisk shows clear cytoplasm cells and black asterisk shows representative polysaccharide intracellular deposits (*iii*). **D.** Highlighted differences observed along the GBM18, GBM27 and GBM38 passages in culture: neurosphere architectural reorganization (*i*), mitochondrial crest architectural loss (*ii*) and double membrane structures (*iii*).

In order to examine the stability of CSC cultures, together with live-cell functions, we first isolated eleven CSC-enriched cultures derived from fresh surgical human GBM samples and cultured them under serum-free conditions [23]. As such, they grew as expandable sphere-like cultures showing different growing features under the optical microscope, based on which they were grouped into three clusters (Supplementary Table S1). One representative culture for each cluster was chosen for further analysis, and their CSC properties were characterized within the first 2 passages (see Supplementary Section). GBM18 grew as spheres attached to the surface that eventually detached (Figure 1A, *i*), GBM27 grew as spheres in suspension (Figure 1B, *i*) and GBM38 grew as an attached monolayer of cells in combination with spheres that eventually detached from the plate (Figure 1C, *i*). Then, with the aim of studying the evolution of CSCs *in vitro* right after their isolation, we subcultured them for up to 20 passages (18 months) without freeze-thaw cycles (Supplementary Figure S5).

### Cell morphology of CSCs and tumourspheres remains stable along passages

We first examined the sphere surface and observed that all three CSC cultures displayed different cellular projection patterns that remained unchanged for all passages studied (Figure 1A, *ii*, 1B, *ii* and 1C, *ii*). Afterwards, we wondered whether the cell organization within the spheres also followed reproducible patterns. Interestingly, for all three CSC cultures, we found the presence of extracellular matrix (Figure 1A, *iii* and *iv*, 1B, *iii* and *iv* and 1C, *iii* and *iv*) and, along the number of passages, we observed an increasing number of spheres displaying wider intercellular space and a higher number of cell membrane projections, some of which interlaced, increasing the membrane surface per cell and contributing to the maintenance of the sphere architecture (Figure 1D, *i*).

Next, the spheres were processed to carry out a comparative study of cell morphology intra- and inter-CSC culture. Serial semithin sections showed significant inter-CSC culture differences but a high grade of intra-CSC culture homology. We observed that the presence of multilobular and polymorphic nuclei was a common feature for all of them. Additionally, dividing cells were observed in both the peripheral regions and the center of the tumourspheres (Supplementary Figure S6). Ultrathin sections revealed that most cells displayed the presence of differential inter-CSC culture abnormal inclusions. GBM18 showed inclusions compatible with polysaccharide deposits (Figure 1A, *v*), GBM27 showed an important number of electron dense inclusions (Figure 1B, *v*) and GBM38 a combination of polysaccharide, electron dense and lipid inclusions (Figure 1C, *v*). Finally, we also observed the alteration of the mitochondrial architecture along the passages (Figure 1A, *vi*, 1B, *vi*, 1C, *vi* and 1D, *ii*) and the presence of double membrane structures in late passages (Figure 1D, *iii*). Interestingly, the cell morphology remained mostly unchanged for all three CSC cultures along the passages.

### CSC-enriched cultures increase their stemness stage along the passages

**Figure 2:**
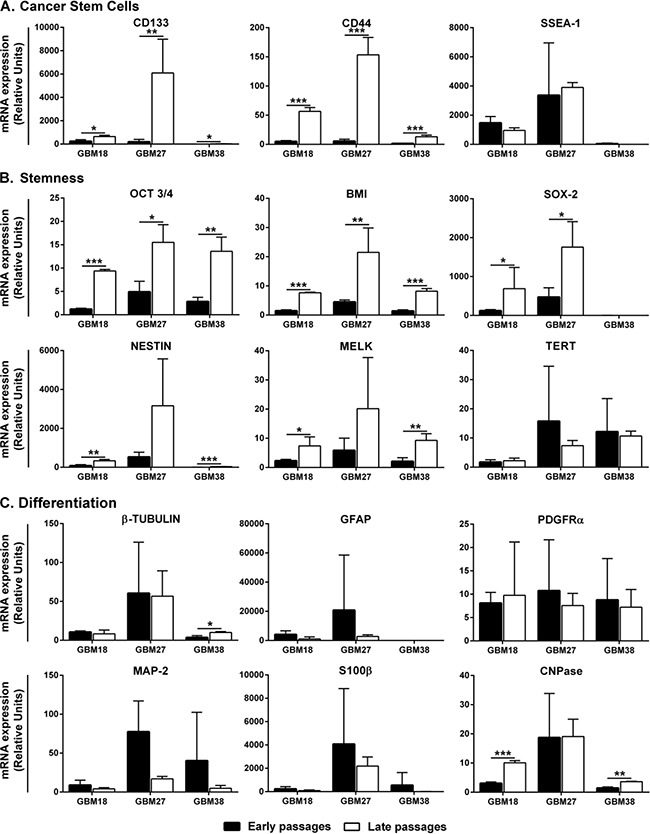
Gene expression analysis at early and late passages of CSC-enriched cultures **A.** Cancer Stem Cell markers. **B.** Stemness markers. **C.** Differentiation markers. Keywords: black columns represent early passages; white columns represent late passages; for every comparative analysis, the sample with lower mRNA gene expression is given the value 1. Error bar represents Standard Deviation. *: P ≤ 0.05; **: P ≤ 0.01 and ***: P ≤ 0.001.

Once we have discarded relevant changes in cell morphology within the tumorspheres, we looked into the mRNA expression of CSC markers. We found that cells in late passages from GBM18, GBM27 and GBM38 expressed higher mRNA levels of *CD133* and *CD44* while those of *SSEA1* remained unchanged (Figure 2A). Consistently, when we analysed the mRNA expression of stem cell markers such as *OCT3/4*, *BMI* and *SOX2* we found increasing values in late passages with the only exception of *SOX2* for GBM38, whose expression level remained low and even along the passages. Additionally, the mRNA expression level of adult neural stem cell markers, such as *NESTIN* and *MELK*, also increased in late passages, while the expression of *TERT* remained high and unchanged (Figure 2B). As the results suggested an increased stemness stage of CSC-enriched cultures along the passages, we also looked into the mRNA expression of differentiation markers for the three main neural lineages. We observed that astrocyte markers (*GFAP* and *S100β*) remained unchanged along the passages for all three CSC-enriched cultures studied. The mRNA expression of neuronal markers remained equal for GBM18 and GBM27 with a little increase of *β-III-Tubulin* in late passages of GBM38. Moreover, we did not observe significant changes in transcript levels of the early oligodendrocyte marker *PDGFRa*, while *CNPase* increased in late passages for GBM18 and GBM38 (Figure 2C).

### Chromosomal instability collaborates with *in vitro* evolution of CSC-enriched cultures

Chromosomal instability is a hallmark of tumor cells, including gliomas, and the main cause of genomic alterations throughout the course of the disease. In order to evaluate the occurrence of genomic alterations in CSCs isolated from surgical samples, over their passages in culture, we carried out sequential Comparative Genomic Hybridization (CGH) assays on GBM18, GBM27 and GBM38. First, we wondered whether the chromosomal instability might be generated by the isolation and culture procedures or due to an inherent feature of CSCs. To rule out the first possibility, we set out to investigate the presence of genomic alterations in Neural Stem Cells (NSCs) isolated from surgical samples of human adult brain - normal counterpart of CSCs - under the same isolation and culture procedures used for CSCs. The analysis of CGH data showed the absence of genomic alterations in NSCs along the first 7 passages, when the cells became senescent (Supplementary Figure S7). These results demonstrated that our isolation and culture procedures did not generate chromosomal instability in human adult NSCs displaying a normal genetic background.

**Figure 3:**
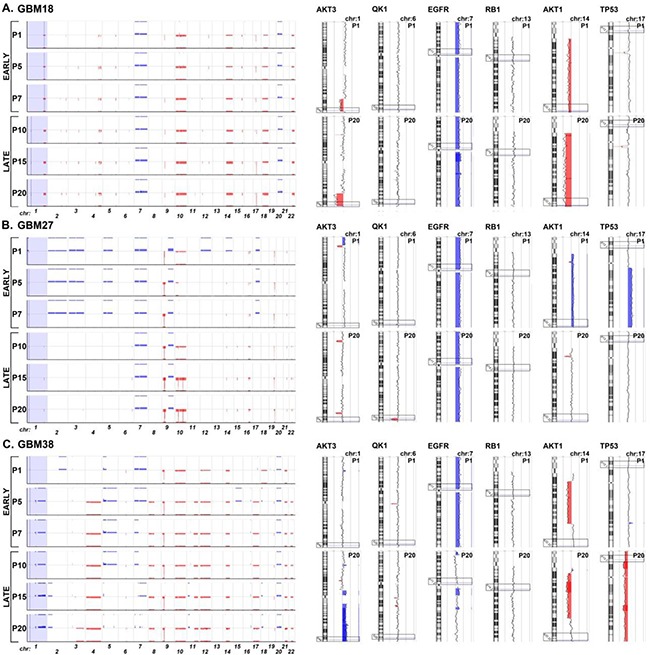
Graphical representation of chromosomal imbalances detected in GBM18, GBM27 and GBM38 CSC-enriched cultures along the 20 first passages in culture after isolation from surgical samples **A.** GBM18. **B.** GBM27. **C.** GBM38. The X-axis represents the chromosomes, while the Y-axis represents the normalized log2 Cy5(CSC)/Cy3(healthy control) fluorescence intensity thresholds -1 (loss(red)) and 1 (gain (blue)), respectively. The genes *AKT3*, *QK1*, *EGFR*, *RB1*, *AKT1* and *TP53*, frequently altered by DNA gains or losses in human gliomas, are shown as ideograms at passage 1 and 20. The clear boxes indicate the location of the gene. Blue represents DNA gains and red DNA losses.

Then, we focused on the analysis of CGH data from all three CSC-enriched cultures – GBM18, GBM27 and GBM38- at passages 1, 5, 7, 10, 15 and 20. As expected, we observed genomic alterations due to DNA losses and gains in all three CSC-enriched cultures. Interestingly, genomic alterations identified in GBM18 remained unchanged for all passages assayed while GBM27 and, to a greater extent, GBM38 displayed chromosomal instability events along the passages (Figure 3A-3C).

In general, DNA losses outnumbered gains in all three CSC-enriched cultures. GBM18 displayed genomic alterations in 17 somatic chromosomes, including 3 DNA gains in chromosomes 3, 7 and 20, and 25 DNA losses in 15 different chromosomes (Figure 3A and Supplementary Table S4). GBM27 also displayed genomic alterations in 17 somatic chromosomes, including 11 gains in 10 chromosomes, although the total number of gains came down to 6 after the first passage. Additionally, this CSC-enriched culture exhibited a total of 26 DNA losses in 11 chromosomes along the 20 passages; being chromosomes 10 and 19 the most affected, with 6 and 5 DNA losses, respectively (Figure 3B and Supplementary Table S5). Finally, GBM38 displayed genomic alterations in all somatic chromosomes. We detected a total of 22 DNA gains in 11 chromosomes, and 28 DNA losses in 17 chromosomes, along all passages (Figure 3C and Supplementary Table S6).

Next, we wondered whether the most recurrent sites of DNA copy-number aberration described in glioma samples, containing a putative oncogene or tumor suppressor gene [24], might be also altered in CSC-enriched cultures. To address this question, we analysed the location of 19 statistically significant DNA gains and 20 DNA losses identified previously from 543 GBM solid samples by TCGA [24] (Supplementary Table S7). GBM18 displayed just 3 DNA gains, all of them at chromosome 7, including the oncogenes *EGFR, CDK6* and *MET,* and 2 DNA losses including *AKT1* and *AKT3*, within the set of recurrent DNA gains. On the contrary, this CSC-enriched culture showed 10 DNA losses (50%) and no DNA gains within the set of recurrent DNA losses (Figure 3A and Supplementary Tables S4 and S7). Interestingly, GBM27 and GBM38 displayed changes on DNA gains and losses throughout the passages, affecting the location of both oncogenes and tumor suppressors. GBM27 showed DNA gains affecting the location of 14 genes within the set of recurrent DNA gains at first passage, although half of them involved less than 50% of the cells. However, after the fifth passage, many DNA gains disappeared, remaining those that affected the location of *MYCN, SOX2, EGFR*, *CDK6*, *MET* and *GRB2*. Nonetheless, after the tenth passage, *MYCN* and *GRB2* DNA sequence gains persisted in less than 50% of cells. Unexpectedly, this CSC-enriched culture displayed 6 DNA gains and just 3 DNA losses within the set of recurrent DNA losses at first passage, although the DNA gains reduced to 5 after the fifth passage, affecting the location of *LSAMP, 3q29* and *NF1*, which actually involved less than 50% of cells after the tenth passage. DNA losses affecting the location of *CDKN2A/B*, *PTEN* and 19q13.33, for all passages, and *QKI*, 10q26.3 and 15q14, in less than 50% of the cells, after the tenth passage were also detected (Figure 3B and Supplementary Tables S5 and S7). Finally, GBM38 showed DNA losses affecting chromosomes 4 and 12 in all passages, and chromosome 17 after the fifth passage, within the set of recurrent DNA gains. These DNA losses matched the location of *FGFR3, PDGFRa, CCND2, CDK4, MDM2* and *GRB2*. Surprisingly, DNA sequence gains affecting the location of *EGFR, CDK6* and *MET* at chromosome 7 disappeared after the tenth passage, while others affecting *AKT3* and *MDM4* at chromosome 1 appeared from the fifth passage on. Consistent with this, the same variability was observed within the set of recurrent DNA losses. We detected 4 DNA gains at the first passage in chromosomes 3, 15 and 22, all of them affecting less than 50% of the cells. However, 3 of these DNA gains disappeared after the fifth passage and the last one after the tenth passage. Interestingly, 2 new DNA gains, affecting chromosome 1, appeared at passage 5 in 100% of the cells and remained along the 20 passages under study. We also observed the presence of 4 DNA losses in all passages, affecting the chromosome 9 (*CDKNA/B*), 10 (*PTEN* and 10q26.3) and 14 (*NPAS3*). Surprisingly, the DNA sequences gained at the first passage, affecting chromosome 22 in less than 50% of the cells, were consistently lost in 100% of the cells from the fifth passage on, and the same happened for the 2 DNA gains affecting the chromosome 3 at the first passage, which turned out to be lost at the last passage. Finally, to add on the high chromosomal instability of this CSC-enriched culture, we found 2 more DNA losses, affecting the chromosome 13 (*RB1* and 13q22.1) in less than 50% of the cells, between the passages 7 and 10 (Figure 3C and Supplementary Tables S6 and S7).

### CSC proliferation and duplication times display fluctuations along the passages

**Figure 4:**
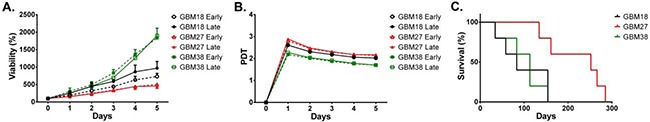
Viability of early and late passages of CSC-enriched cultures and average survival of mouse models of their respective brain tumor xenotransplants **A.** Viability assay for early and late passages of GBM18, GBM27 and GBM38. **B.** Duplication time for early and late passages of GBM18, GBM27 and GBM38. **C.** Kaplan-Meier survival curves for mice xenotransplanted with GBM18, GB27 and GBM38.

We next wondered whether the observed changes in morphology, chromosomal instability and differentiation state along the passages in culture, might influence the proliferation ratio of CSC-enriched cultures. To address this question, we carried out viability assays with early and late passages cultured *in vitro* for up to 5 days. In general, we found no significant differences in the percentage of viable cells between early and late passages of GBM27 and GBM38. Interestingly, we did observe significant differences between early and late passages of GBM18 (P < 0.01) (Figure 4A). GBM27 displayed the higher duplication time followed by GBM18 and GBM38, which showed the highest proliferation rate *in vitro* (Figure 4B). Then, we wanted to know whether the proliferation rate *in vitro* correlated with survival of mouse models of brain tumor xenotransplanted with these CSCs. Consistently, we found that the overall survival of xenotransplanted mice with GBM18 and GBM38 was quite similar, around 100 days, while those mice xenotransplanted with GBM27 displayed longer overall survival with an average of 220 days (Figure 4C).

### *In vivo* migration and dissemination patterns of CSCs remain unchanged along passages

**Figure 5:**
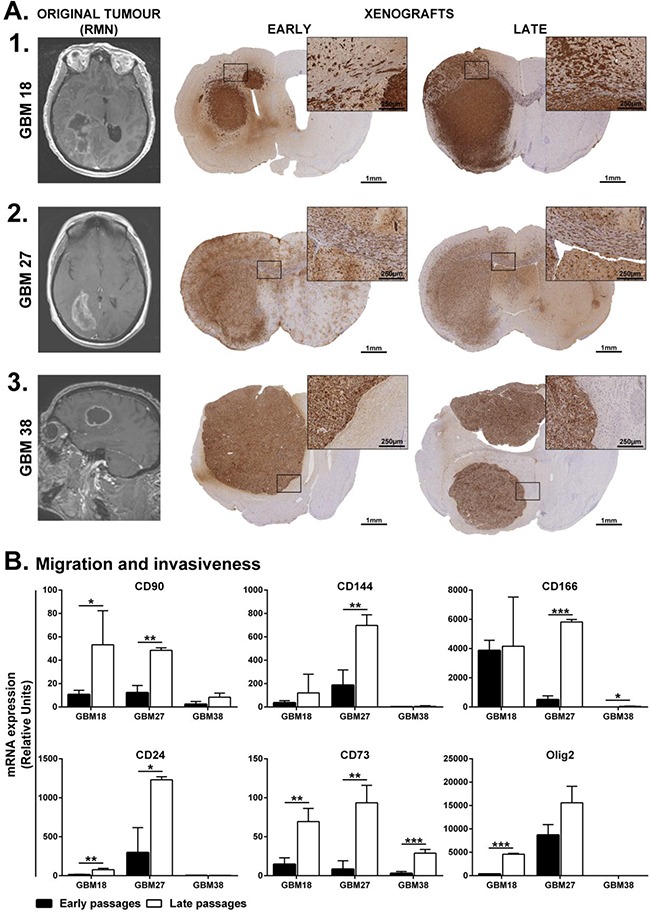
Brain dissemination patterns of early and late CSCs isolated from human samples **A.** MRI from the original tumor and mouse model of brain tumor xenotransplanted with GBM18 (A1), GBM27 (A2) and GBM38 (A3) at early and late passages. GBM18 and GBM27 are clinically defined as highly disseminated while GBM38 as nodular. Squared regions are shown at higher magnification. **B.** Gene expression analysis of migration and invasiveness markers at early and late passages of CSC-enriched cultures. Keywords: black columns represent early passages; white columns represent late passages; Error bar represents Standard Deviation. *: P ≤ 0.05; **: P ≤ 0.01 and ***: P ≤ 0.001.

To evaluate the ability of these CSC-enriched cultures to recapitulate the original tumor features, we carried out orthotopic transplantations within the striatal brain of adult nude mice. Staining with anti-human Vimentin, to expose human cells, revealed the *in vivo* migration and invasion capacity of these three CSC-enriched cultures. At the time of diagnosis, GBM18 and GBM27 were clinically described as highly disseminating, while GBM38 was described as nodular (Figure 5A, 1-3). Consistent with clinical description, GBM18 formed a nodular-like tumor mass; however, the nodular boundaries were not well defined. The cells migrated out of the nodular tumor mass and invaded the neighbouring tissues, reaching the contralateral hemisphere at both early and late passages (Figure 5A, 1). Interestingly, GBM27 CSCs disseminated through the mouse brain and invaded the contralateral hemisphere at both early and late passages (Figure 5A, 2). On the contrary, GBM38 CSCs remained within the ipsilateral hemisphere forming a nodular-like tumor mass, with well-defined boundaries, also at both early and late passages (Figure 5A, 3). We found GBM18 CSCs surrounding blood vessels in the striatum, but not GBM27 CSCs. Both GBM18 and GBM27 CSCs seemed to accumulate along the ventricular wall and use the myelin fiber tracks within the striatum and the *corpus callosum* to migrate (Figure 5 and Supplementary Figure S8). In order to find a molecular signature that explained this histological finding, we studied the mRNA expression of genes related to migration and invasiveness. Consistently, the mRNA expression of *CD90*, *CD144*, *CD24*, *CD73* and *OLIG2* was higher in GBM27 followed by GBM18 and GBM38. Interestingly, the expression levels of *CD90*, *CD144*, *CD166*, *CD24* and *CD73*, were significantly increased in the late passages of GBM27, as well as *OLIG2* to a lesser extent. Similar results were observed when we analysed *CD90*, *CD24*, *CD73* and *OLIG2* for GBM18 and *CD166* and *CD73* for GBM38. However, the higher mRNA expression of migration and invasiveness markers at late passages did not translate into an increased migration of CSCs *in vivo* (Figure 5B).

### The CSCs response to drugs evolves in vitro

**Figure 6:**
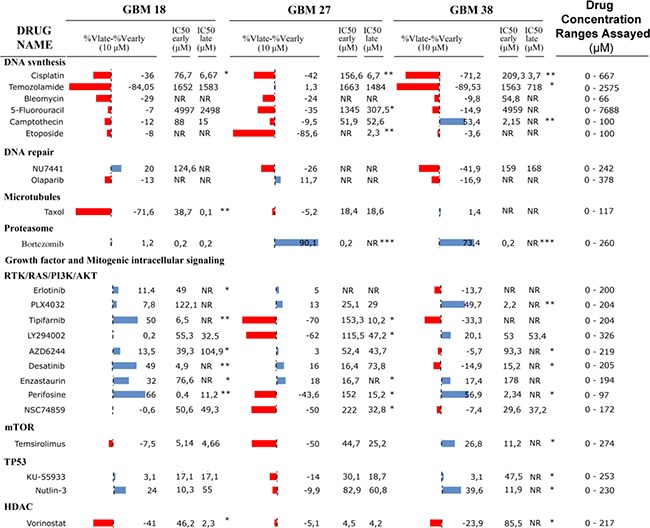
Drug sensitivity panel of early and late CSCs isolated from human samples For every CSC-enriched culture viability differences (% viability at late passages - % viability at early passages), as well as the viability at IC_50_ for early and late passages are shown. Keywords: red bars mean that early passages are more sensitive than late passages; blue bars mean that early passages are less sensitive than late passages; asterisks represent differences between early and late IC_50_ by * (one), ** (two) or *** (three) orders of magnitude.

Once we confirmed that all CSC-enriched cultures, regardless the passage in culture, reproduced the migration and dissemination patterns *in vivo*, we wanted to know whether the time in culture influenced their sensitivity to a panel of drugs currently used in both clinical trials and clinical practice against GBM. To address this question, we carried out functional viability assays with three different early and late passages for all CSC-enriched cultures (Figure 6). The IC_50_ as well as the percentage of cell survival at 10 μM were calculated by lineal interpolation to compare drug sensitivity between early and late passages. We found that all three CSC cultures showed significant differences between early and late passages in both the IC_50_ and the viability at 10 μM for at least 20% of the assayed drugs. Interestingly, late passages were significantly more sensitive to cisplatin. In the same line, we observed that all three CSC cultures displayed higher sensitivity to drugs interfering with DNA synthesis at late passages, with the only exception of camptothecin for which GBM38 showed an IC_50_ at least 10 times higher at late passages. However, there were no differences between early and late passages when we assayed drugs that interfered with DNA repair. GBM18 and GBM27 displayed sensitivity to taxol but there were important differences between both CSC cultures. While the IC_50_ between early and late passages of GBM27 was quite similar, late passages of GBM18 displayed an IC_50_ more than 50 times lower than early passages. The three CSC cultures were sensitive to bortezomib; however, GBM27 and GBM38 were significantly more sensitive at early passages. Then, we assayed a collection of drugs targeting growth factors and cell signalling pathways. We observed an IC_50_ more than 10 times lower at early passages of GBM18 for tipifarnib, desatinib and perifosine, and the opposite for vorinostat. GBM27 also displayed significant differences between late and early passages for perifosine, enzastaurin and tipifarnib. Finally, we found significant differences between early and late passages of GBM38 for PLX4032 and to a lesser extent for desatinib, perifosine, temsirolimus and Nutlin-3 (Figure 6).

## DISCUSSION

The percentage of surgical samples from which we were able to isolate expandable CSC cultures, as well as the different growth patterns observed, is consistent with previous works [11, 17, 18]. These patterns apparently did not change along the passages when observed under the OM. However, a closer analysis under the EM revealed morphological changes common to all three CSCs, as well as more specific features for each of them. For all analyzed CSC lines, TEM confirmed common architectural reorganization of spheres and a higher number of disrupted mitochondrial crests over the passages. The widening of intercellular spaces, together with the increase of membrane surface per cell, probably played a role in permitting the access of fresh nutrients to internal cells as well as the clearance of residual metabolites. Additionally, the alteration of mitochondrial cytoarchitecture might be compatible with a progressive selection of glycolysis for glucose metabolism *in vitro* [25]. In this regard, the ability of CSCs isolated from human gliomas to use multiple pathways for energy production has been recently demonstrated, suggesting that CSCs might be refractory to treatments targeting individual metabolic pathways [26]. Moreover, we observed abnormal deposits of different types of macromolecules in all three CSC lines, probably as a result of altered metabolic pathways. In this regard, the deposit volume might be related to the differentiation stage; the clear cytosolic cells, found within the sphere, might thus correspond to the most undifferentiated CSC subpopulation. Interestingly, for all analysed CSC lines, the morphology of most cells within the spheres was highly homogeneous, which suggests a clonal origin of these cells.

In line with the homogeneous cell morphology within each CSC line, the analysis of specific molecular marker expression along the passages revealed a striking similarity between the analysed CSC lines. These results support the uniform presence of differentiated cells within the tumorspheres along the passages, and are consistent with the observation that cytosolic cells were overloaded with aberrant deposits. Furthermore, these results suggest a positive selection of CSCs with more prominent stemness stage. Our data thus confirm previous genomic analyses of CSC lines from different passages, which showed that CSC biological replicates grouped together when compared with other samples [15, 17, 27]. However, the relative expression of specific biomarkers between the passages has not been previously reported.

In the last few years, several groups have published a collection of data demonstrating the existence of intra-tumor heterogeneity in human glioblastoma [12, 13, 28]. Consistent with these reports, we found that most chromosomal alterations affected 100% of the cell populations along the 20 passages after isolation. This finding, together with the morphologically homogeneous intra-tumoral CSCs, points to a clonal origin for the tumor cells present in primary cultures. Interestingly, our CGH analysis shows that the current protocols for CSC enrichment and culture, under serum-free conditions, randomly generate both homogeneous and heterogeneous populations of CSCs. We observed DNA gains, affecting less than 50% of the population, in the first passage of GBM27 and GBM38 cells that disappeared a few passages later, which might suggest the presence of clones less prone to survival or competitiveness than other clones *in vitro*. Additionally, DNA gains and losses affecting less than 50% of the population showed up at different passages, and then either disappeared or remained underrepresented in the total population. Finally, for these two CSC-enriched cell lines, we also observed new DNA gains and losses, affecting 100% of the population, at different passages. On the contrary, CGH analysis showed no significant changes for the GBM18 homogenous population. All together, these results suggest that a heterogeneous population of CSC-enriched cell lines evolve *in vitro* as they do *in vivo*, although essential differences in microenvironment cues probably drive the evolution process in different directions. We found that CSC-enriched cultures displayed similar duplication times, meaning that the chromosomal instability and gene expression variability observed along passages did not translate into significant proliferation changes *in vitro*. Interestingly, mouse models of brain tumor xenotransplated with GBM27, that displayed the higher duplication time *in vitro*, showed the longest average survival. This result suggests a link between low proliferation *in vitro* and *in vivo*, however other parameters like the brain dissemination pattern, may also influence and more experiments with a higher number of CSC- lines will be needed to clarify this question.

Notably, the dissemination pattern of CSCs through the parenchymal brain in mice remained unchanged along the passages - resembling the original tumors -. This finding suggests the presence of either specific mutations or, most probably, coordinated molecular alterations, passed on from the original clone, responsible for the migration and dissemination. Consistently, other authors observed no changes in the dissemination patterns *in vivo* from different passages of CSCs [11, 17]. However, considering that CSCs also evolve *in vitro*, it is important to note that new alterations affecting essential molecules in migration pathways along the passages *in vitro* might modify the dissemination pattern *in vivo*. Importantly, we showed three different patterns of dissemination *in vivo* for CSCs isolated from human glioma samples. GBM27 displayed a highly disseminating pattern, longer overall survival and higher expression of *OLIG2*, consistent with the CSC type I described by Günther *et al.*, and the proneural phenotype [29]. On the contrary, GBM18 and GBM38 displayed a nodular dissemination pattern and similar overall survival, which resemble the CSC type II , and the mesenchymal phenotype [16, 17, 29, 30]. However, GBM18 showed irregular boundaries, with cells migrating out of the main tumor mass, and significantly higher *OLIG2* expression than GBM38. Therefore, the disseminated, nodular and semi-nodular preclinical *in vivo* models of GBM constitute three separated tumor behaviours that might need different therapeutical approaches.

Supporting the idea of *in vitro* evolution of CSC-enriched cultures, we also observed significant variations in the IC_50_ for a number of drugs along the first 20 passages in culture. These variations affected both homo (GBM18) and heterogeneous (GBM27 and GBM38) CSC-enriched cultures. The *in vitro* DNA gains and losses might thus not be enough to explain the drug-sensitivity variations, and genetic and/or epigenetic alterations along the passages might also play a role. These results suggest that reliable functional experiments need to be performed on well-characterized CSC-enriched cultures within a limited number of passages.

Altogether, the CSC-enriched cultures from surgical samples are now the most reliable preclinical models of human high-grade glial tumors. However, these models are far from mimicking the original tumors. Indeed, these cultures evolve *in vitro* acquiring new features as a result of their intrinsic instability and microenvironment cues. Therefore, in order to take advantage of the whole potential of these preclinical tumor models to develop new therapeutic approaches, it will be essential to isolate a large number of CSC-enriched cultures and characterize them not only in the context of their original tumors but also their evolution *in vitro*.

## MATERIALS AND METHODS

### Isolation and culture of CSCs from human GBM samples

Cancer stem cells were isolated from human fresh GBM samples. Tissue samples were obtained from patients operated at the Neurosurgery department (Hospital la Fe, Spain). Permission to use this material was obtained from the ethical review board in Hospital la Fe and Principe Felipe Research Center, and written informed consent was obtained from patients. GBM CSCs and normal brain parenchyma cells were cultured in media containing: DMEM/F-12 (Gibco, 11039), Non Essential Amino Acids (10mM; Gibco, 11140), Hepes (1M; Gibco, 15630), D-Glucose (45%; Sigma, G8769), BSA-F5 (7,5%; Gibco, 15260), Sodium Pyruvate (100mM; Gibco, 11360), L-Glutamine (200mM; Gibco, 25030), Antibiotic-Antimycotic (100x; Gibco, 15240), N_2_ Supplement (100x; Gibco, 17502), Hydrocortisone (1μg/μl; Sigma, H0135), Tri-iodothyronine (100μg/ml; Sigma, T5516), EGF (25ng/μl; Sigma, E9644), bFGF (25ng/μl; Sigma, F0291) and Heparin (1μg/μl; Sigma, H3393).

### Tumorsphere morphological analysis

To evaluate possible changes in both the sphere architecture and cell morphology, we separated 12 spheres of 150 μm diameter from each CSC culture at different time points (Supplementary Figure S5) and further analysed them by optical microscopy (OM), Screening Electronic Microscopy (SEM) and Transmission Electronic Microscopy (TEM). Tumorspheres were fixed with 3.5% glutaraldehyde (Electron Microscopy Science, Hatfield, USA) for 1 h at 37°C. Afterwards, they were embedded in 3% agar drops and postfixed with 1% osmium tetroxide (Sigma), rinsed, dehydrated, and embedded in araldite (Durcupan, Sigma). For brain tissue analysis, at the appropriate time points, mice were deeply anesthetized with an intraperitoneal injection of Ketamine (100mg/kg) and Medetomidine (0,5mg/kg), and transcardially perfused with a 0.9% NaCl solution followed by a 2% paraformaldehyde/2.5% glutaraldehyde solution (PFA/GA, Electron Microscopy Sciences, Hatfield, PA) in PBS. Brains were removed, post-fixed in PFA/GA overnight and rinsed in cold PBS (5×10 min). After fixation, brains were cut into 200 μm sections on a vibratome (Leica VT-1000), rinsed, dehydrated and embedded in araldite (Durcupan, Sigma). For both types of samples, tumorspheres and brain tissue slides, semithin sections (1.5 μm) were cut with a diamond knife and lightly stained with 1% toluidine blue (Panreac, Barcelona, Spain). Semithin sections were detached from the glass slide by repeated freezing (liquid nitrogen) and thawing and re-embedded in an araldite block. The block with semithin sections was cut in ultrathin (0.05 μm) sections with a diamond knife, stained with lead citrate, and examined under a Tecnai Spirit Electron Microscope (FEI). Photographic images were taken with a Morada camera (Soft Image System, Munster, Germany).

### RT and QRT-PCR

For RT-PCR, total RNA was isolated using RNeasy Mini or Micro kit (QIAGEN) following the manufacturer's recommendations. One μg of RNA was used for cDNA synthesis (High-Capacity cDNA Reverse Transcription Kit; Applied BioSystems). Samples were amplified with specific primers and the Paq5000 polymerase (Stratagene), in a Mastercycler (Eppendorf). QRT-PCR was run in a LightCycler 480 Instrument (Roche). For each experiment, controls were performed in which reverse transcriptase was omitted from the cDNA reaction mixture and template DNA was omitted from the PCR mixture.

### Cancer stem cell differentiation

Cells were plated for 10 days on 8-well chamber slides (Nalgene Nunc, 177402) coated with Matrigel Basement Membrane Matrix (2mg/ml; BD, 356234) in growth factor-free media supplemented with 10% FBS. Cells were then fixed in 4% paraformaldehyde.

### Immunocytochemistry

Cells were fixed in 4% paraformaldehyde for 20 min, washed with PBS and incubated in 0.2% Triton X-100/PBS for 20min at 37°C and 10min at RT. Cells incubated with BrdU were treated with 1N HCl for 30 min at 37°C and neutralized in 0.1M borate buffer, pH 8.5 for 5min at RT. After treatment with 5% normal goat serum/0.1% Triton X-100/PBS for 15min, cells were incubated overnight at 4°C with the following primary antibodies: BrdU (mouse monoclonal, 1:100, Dako), GFAP (rabbit polyclonal, 1:1000, Dako), SOX2 (goat, 1:50, Chemicon), Nestin (rat, 1:100, Chemicon), Tuj1 (chicken, 1:500, Sigma) and CNPase (mouse monoclonal, 1:400, Abcam). Cells were then incubated 1h with the corresponding Alexa Fluor conjugated secondary antibodies (1:500) and treated with DAPI (1:1000, Sigma) for 15min. Coverslips were mounted in Fluorsave Reagent (Calbiochem, 345789).

### Immunohistochemistry

Formalin-fixed paraffin-embedded sections were stained (as per the manufacturer's staining protocol) with the Bond Polymer Refine Detection Kit on a Bond-max™ fully automated staining system (Leica Microsystems GmbH, Germany), using a mouse monoclonal antibody against human Vimentin (1:500, Santa Cruz Biotechnology).

### Genomic DNA microarray

Genomic DNA was quantified by spectrophotometry (NanoDrop ND1000, NanoDrop Technologies, Wilmington, Delaware USA). Integrity of DNA was assessed by 0.8% agarose gel electrophoresis. Non-amplification labeling of DNA (direct method) was obtained following the ‘Agilent Oligonucleotide Array-Based CGH for Genomic DNA Analysis’ protocol Version 4.0 (Agilent Technologies, Palo Alto, California USA. p/n G4410-90010). 500 ng of experimental and pool female reference genomic DNA samples were fragmented in a restriction digestion step. Digestion was confirmed and evaluated by DNA 7500 Bioanalyzer assay. Cyanine 3-dUTP and cyanine 5-dUTP were used for fluorescent labeling of test and reference digested gDNAs respectively, using the ‘Agilent Genomic DNA Labeling Kit PLUS’ (Agilent p/n 5188-5309) according to the manufacturer's instructions. Labeled DNA was hybridized with Human Genome CGH Microarray 44K (Agilent p/n G4426B-014950) containing 43,000+ coding and noncoding human sequences. Arrays were scanned in an Agilent Microarray Scanner (Agilent G2565BA) according to the manufacturer's protocol and data extracted using Agilent Feature Extraction Software 9.5.3.1 following the Agilent protocol CGH-v4_95_Feb07 (‘Lowess Only’ normalization correction dye bias method instead of ‘Linear Only’) and the QC Metric Set CGH_QCMT_Feb08.

### Xenografts

All mouse experiments were approved by and performed according to the guidelines of the institutional animal care committee of Principe Felipe Research Center in agreement with the European Union and national directives. An average of 75.000 cells were stereotactically injected into the striatum of the right brain hemisphere (0 mm anterior and 2,5 mm lateral to the bregma; 3,5 mm intraparenchymal) of 9 week-old NUDE mice (Charles River Laboratories). Mice were euthanized when they presented neurological symptoms or a significant loss of weight.

### MTS assays

The viability of early and late CSC-enriched culture *in vitro* as well as their sensitivity to different drugs was assessed using the MTS assay. Briefly, single-cell suspensions of CSCs were plated in a 96-well plate, 3000 cells/well in a final volume of 80 μl/well. For the viability assay, they were allowed to grow up to 5 days and analyzed every 24 hours. For drug sensitivity assays, the cells were allowed to grow and to form spheres for 4 days. Cultures were then treated with 20 μl/well of media (control cells), vehicle (DMSO or water) or increasing concentrations of each drug for 72h. 20 μl/well of MTS (CellTiter 96 AQueous One Solution Cell Proliferation Assay, Promega) was added to the culture media, incubated at 37°C for 3 hours and absorbance was measured at 490 nm. Sensitivity was assessed by comparing the absorbance values of drug-treated cells with vehicle-treated cells and with the absorbance values of control cells (untreated cells) for each treatment group. Each treatment group was repeated in quadruplicate and each experiment in duplicate. Lines were classified as sensitive if their viability decreased or remained unchanged compared with controls.

### Statistical analysis

Statistical analyses were performed using a 2-tailed Student *t* test. Data are presented as means ± standard deviation and were calculated using the software package GraphPad Prism v. 5.0. Statistical values of p > 0.05 were not considered significant.

## SUPPLEMENTARY MATERIALS FIGURES AND TABLES
















