# Supplementary material for: Cancer stem cells from human glioblastoma resemble but do not mimic original tumors after in vitro passaging in serum-free media
Source: Oncotarget. 2016 Aug 29;7(40):65888–901. doi: 10.18632/oncotarget.11676 (PMC5323200; doi:10.18632/oncotarget.11676)
Supplement: Supplementary file 1 [file oncotarget-07-65888-s001.pdf]

# Cancer stem cells from human glioblastoma resemble but do not mimic original tumors after *in vitro* passaging in serum-free media

## SUPPLEMENTARY MATERIALS

### Isolation and characterization of CSC-enriched cultures from human GBM

Cells derived from fresh surgical human GBM samples were isolated and cultured under the same serum-free conditions used for neural stem cells isolated from human adult brain [2]. About 55% of GBM samples (11 out of 20) rendered expandable cell cultures. Consistent with a previous work reported by Günther *et al*., [3] we observed that CSC-enriched cell lines cultured under these conditions showed different growing features that can be grouped into three clusters: spheres attached to the surface that eventually detached (cluster 1), spheres growing in suspension (cluster 2) and attached monolayer of cells in combination with spheres that eventually detached from the plate surface (cluster 3) (Supplementary Table S1). For the purpose of the present work, we randomly took one representative CSC-enriched culture from each group (Figure 2).

To rule out the possibility that these cells were normal progenitor cells, we performed a Comparative Genomic Hibrydization (CGH) analysis on total DNA isolated from the first passage in culture. All three cell lines exhibited loss of heterogeneity (LOH) in chromosome 10 and duplication of chromosome 7, among other chromosomal alterations. All these alterations are hallmarks of glioblastoma (Supplementary Figure S1).

The selected CSC-enriched cultures were grown under differentiation conditions, giving rise to cells expressing astrocyte, oligodendrocyte and neuron markers. We found an important number of cells co-expressing several lineage-restricted markers, which have been previously reported and regarded as another feature of brain tumor cells (Immunocytochemistry assay results for GBM18 shown as example in Supplementary Figure S2). Then, we studied the expression of CSC markers together with stemness and differentiation gene markers. We first designed and validated a collection of primers targeting the selected gene markers (Supplementary Table S2). We observed that GBM18 and GBM27 expressed *CD133*, *SSEA1*, *CD44* and *CD90* genes, all of them reported in the literature to label CSCs subpopulations, in addition of stemness genes also found in adult NSCs. Interestingly, we found no expression of *CD133* and *SSEA1*, and a very low expression of *Nestin* in GBM38 (Supplementary Figure S3). Further analysis of GBM38 CGH showed a LOH at 4p15.32 and 11q21, the chromosomal locations of *CD133* and *SSEA1* respectively (Supplementary Table S3). Finally, we observed equal expression of neuronal markers *MAP-2* and *βIII-Tub* in all three CSC-enriched cell lines while the astrocytic marker *GFAP* was just expressed in GBM18 and GBM27, and oligodendrocyte markers *CNPase* and *PDGFRα* were highly expressed in GBM18 and GBM38 (Supplementary Figure S3). Finally, using Shotgun proteomics, we studied these markers at the protein level. We observed the presence of CD44, Nestin, βIII-Tub, MAP2, and CNPase in all three CSC-enriched cultures. Additionally, we detected SOX2 and GFAP in GBM18 and GBM27 and PDGFRα in GBM18 and GBM38. The presence of GFAP and SOX2, in GBM18 and GBM27 and Nestin, in all three CSCs-enriched cultures, was also confirmed by immunocytochemistry (Supplementary Figure S4).

## SUPPLEMENTARY FIGURES AND TABLES

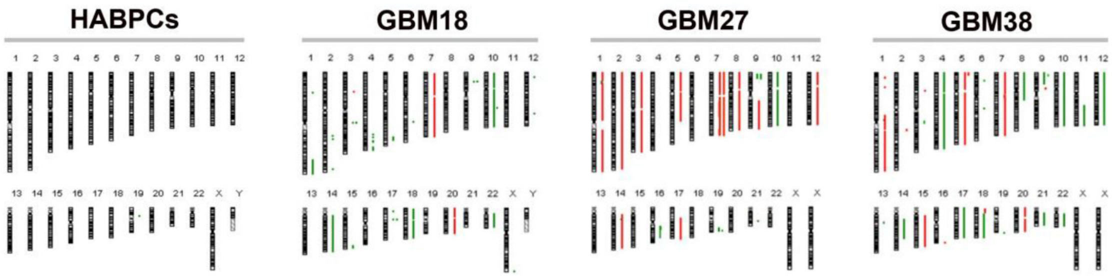


**Supplementary Figure S1: Graphical karyotipe representation of the GBM18, GBM27 and GBM38 CSC-enriched cultures by Comparative Genomic Hybridisation at passage 2.** The chromosomal abnormalities are compatible with GBM. Human Adult Brain Progenitor Cells (HABPCs), isolated from surgical tissue, at passage 1, were used to evaluate the influence of culture conditions on chromosomal instability Keywords: Green line indicates DNA loss and Red line indicates DNA gain.

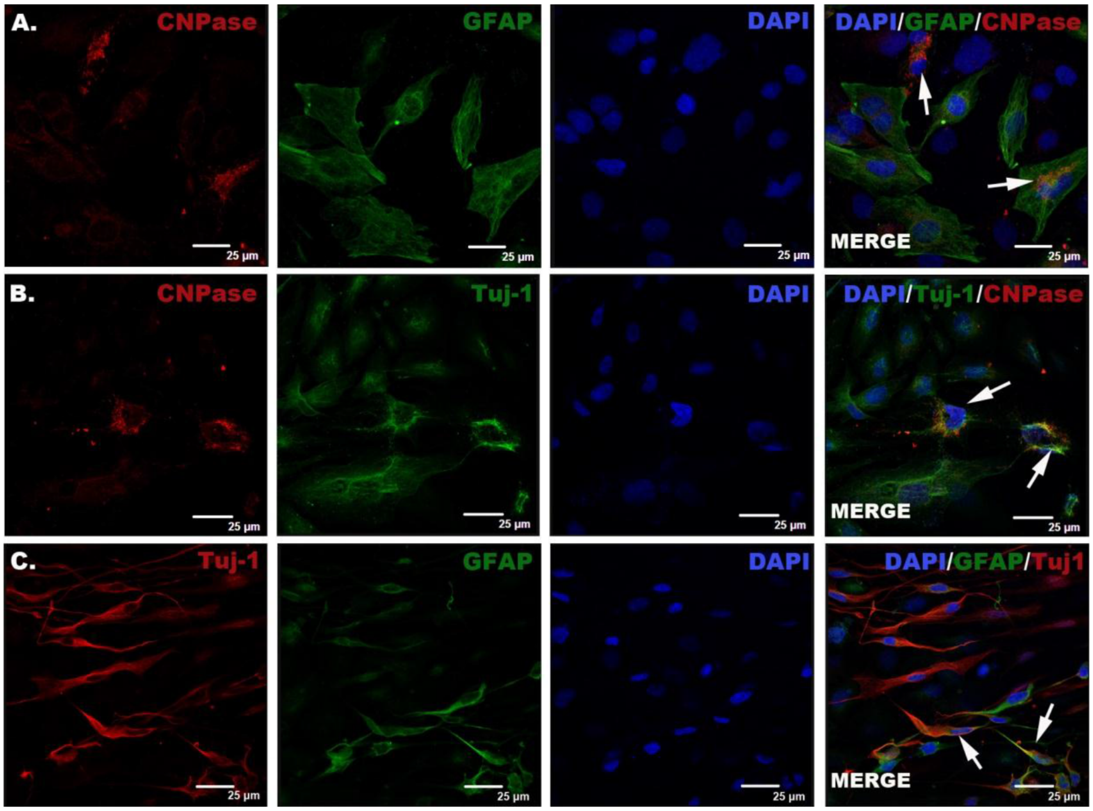


**Supplementary Figure S2: Spontaneous differentiation of CSCs induced by serum addition to culture media for ten days.** The pictures represent the results for GBM18. **A.** GFAP and CNPase co-expression; **B.** Tuj-1 and CNPase co-expression and **C.** Tuj-1 and GFAP co-expression. White arrows represent cells co-expressing markers.

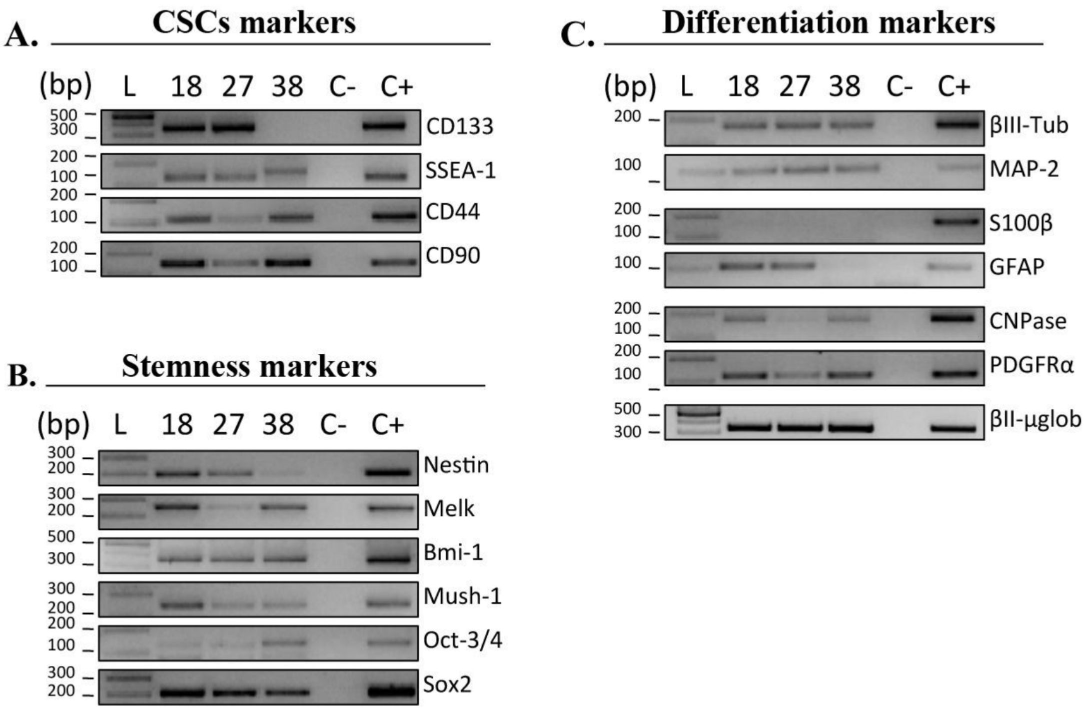


**Supplementary Figure S3: A.** mRNA expression pattern of GBM18, GBM27 and GBM38 for CSC. Stemness **B.** and differentiation markers **C.** The assay indicates presence or absence of the markers.

A.

| Type of marker | Uniprot ACC | Entry Name | Protein Name | Gene Symbol | GBM18 Exclusive unique peptides | GBM18 Total unique peptides | GBM18 Sequence coverage (%) | GBM27 Exclusive Unique peptides | GBM27 Total unique peptides | GBM27 Sequence coverage (%) | GBM38 Exclusive Unique peptides | GBM38 Total unique peptides | GBM38 Sequence coverage (%) |
|---|---|---|---|---|---|---|---|---|---|---|---|---|---|
| CSCs | O43490 | PROM1_HUMAN | Antigen AC133 | PROM1 | - | - | - | - | - | - | - | - | - |
| | P16070 | CD44_HUMAN | CD44 antigen | CD44 | 1 | 8 | 3% | 1 | 5 | 10% | 1 | 5 | 3% |
| | P22083 | FUT4_HUMAN | Fucosyltransferase 4 | FUT4 | - | - | - | - | - | - | - | - | - |
| STEMNESS | Q01860 | PO5F1_HUMAN | Oct-3/4 | POU5F1 | - | - | - | - | - | - | - | - | - |
| | P35226 | BMI1_HUMAN | BMI-1 | BMI1 | - | - | - | - | - | - | - | - | - |
| | P48431 | SOX2_HUMAN | SOX-2 | SOX2 | 1 | 1 | 5% | 2 | 3 | 26% | - | - | - |
| | P48681 | NEST_HUMAN | Nestin | NES | 16 | 49 | 10% | 85 | 49 | 52% | 1 | 1 | 2% |
| | Q14680 | MELK_HUMAN | hMELK | MELKV4 | - | - | - | - | - | - | - | - | - |
| | O14746 | TERT_HUMAN | HEST2 | TERT | - | - | - | - | - | - | - | - | - |
| DIFFERENTIATION | Q13509 | TBB3_HUMAN | Tubulin beta-3 chain | TUBB3 | 0 | 17 | 21% | 3 | 18 | 60% | 1 | 23 | 26% |
| | P14136 | GFAP_HUMAN | GFAP | GFAP | 6 | 16 | 17% | 27 | 14 | 56% | - | - | - |
| | P16234 | PGFRA_HUMAN | PDGFR-alpha | PDGFRA | 2 | 2 | 4% | - | - | - | 1 | 1 | 3% |
| | P11137 | MTAP2_HUMAN | MAP-2 | MAP2 | 1 | 1 | 0% | 4 | 4 | 3% | 2 | 2 | 2% |
| | P04271 | S100B_HUMAN | Protein S100-B | S100B | - | - | - | - | - | - | - | - | - |
| | P09543 | CN37_HUMAN | CNPase | CNP | 4 | 7 | 16% | 17 | 10 | 38% | 2 | 6 | 8% |

B. GBM18 C. GBM27 D. GBM38

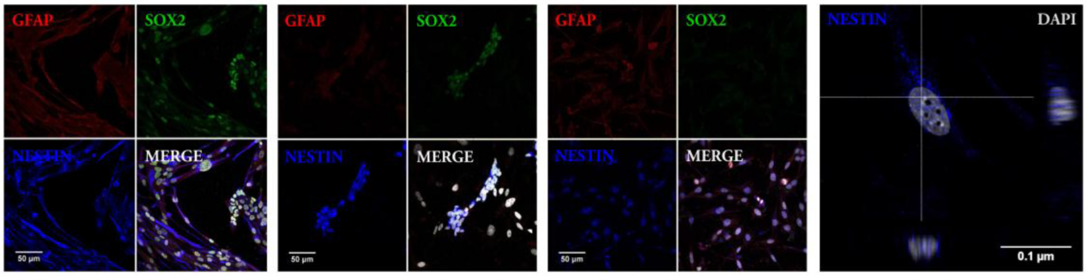


**Supplementary Figure S4: Cancer stem cells, stemness and differentiation markers identified in total proteome of GBM18, GBM27 and GBM38 by shotgun proteomics analysis and confocal microscopy images showing SOX2, GFAP and Nestin immunocytochemistry analysis in CSC-enriched culture cells at late passages.** **A**. Whole cell lysates were digested with trypsin and fractionated by reverse-phase chromatography at basic pH prior to LC-MS/MS (liquid chromatography coupled to tandem mass spectrometry) analysis in a 5600 TripleTOF system (AB SCIEX). Uninterpreted MS/MS data were searched against a human database (UniprotKB, 2013) concatenated with reversed sequences as decoy (containing 73,704 sequences, 36,852 forward sequences), using an in-house licensed Mascot v2.4 search engine. Data analysis was performed using Scaffold (v 4.4.5, Proteome Software), applying a False Discovery Rate (FDR) below 1% at the peptide level. **B**. GBM18. **C.** GBM27. **D.** GBM38. GFAP (red), SOX2 (green), Nestin (blue) and Nuclei (grey). Keywords: *Type of marker*: Classification of protein markers identified by shotgun proteomics; *Uniprot ACC:* accession number of the identified proteins based on Swiss-Prot database; *Entry Name*: the protein ID of the identified proteins based on Swiss-Prot database; *Protein Name*: the name of identified proteins based on Swiss-Prot database; *Gene symbol*: the gene name of the identified proteins based on Swiss-Prot database; *Exclusive Unique Peptides*: the number of different amino acid sequences, regardless of any modification, that are associated with a single protein (as defined by Scaffold software); *Total Unique Peptides*: number of different amino acid sequences that are associated with a specific protein, including those shared with other proteins (as defined by Scaffold software); *Percentage sequence coverage*: the percentage of all the amino acids in the protein sequence that were detected in the sample (as defined by Scaffold software).

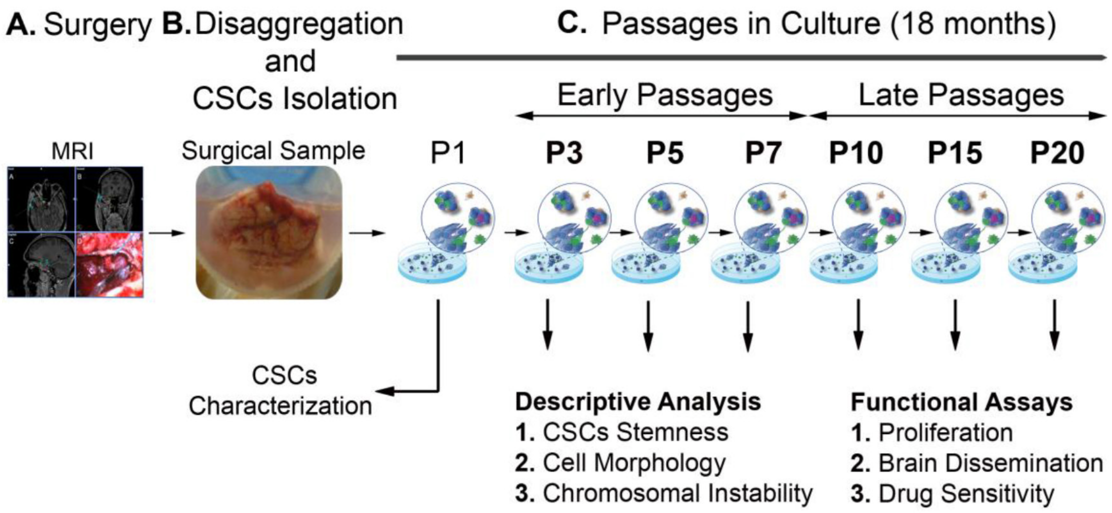


**Supplementary Figure S5: Diagram depicting the experimental design and assays on CSC-enriched cultures isolated from surgical human glioma samples. A.** Standard image of intraoperative MRI indicating the location from where the tumor sample is resected. **B.** Image of regular surgical sample after resection conserved in PBS before processing. **C.** Schematic representation of early and late passages of CSC-enriched culture as well as the different descriptive analyses and assays conducted with the indicated passages. The growth patterns of each CSC culture were conserved along the 20 passages *in vitro*.

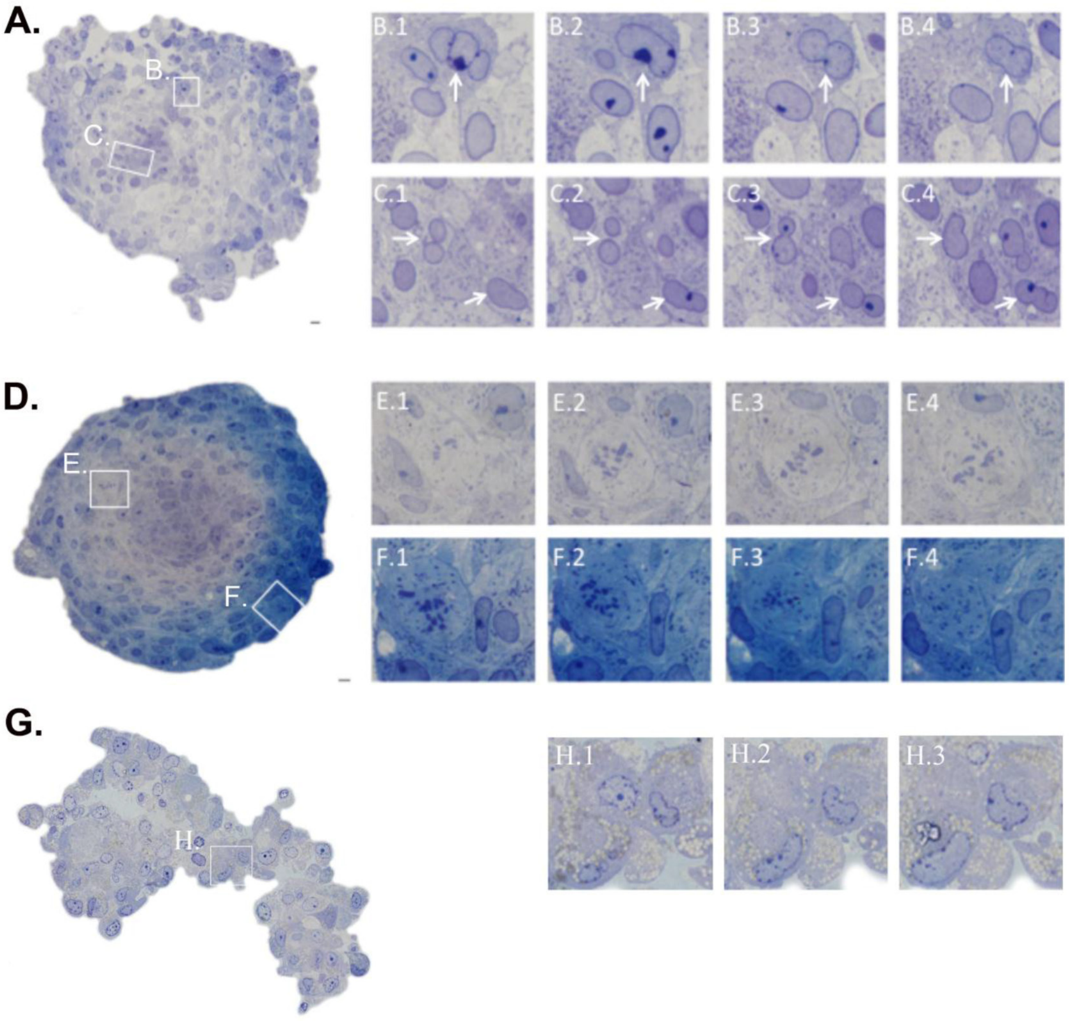


**Supplementary Figure S6: Semithin sections of tumorspheres stained with toluidine blue. A.** GBM18 tumorsphere. **B1-B4** and **C1-C4**. The white squares show polymorphic nuclei revealed by serial images. The white arrows indicate the location of the nuclei; **D.** GBM27 sphere. **E1-E4** and **F1-F4**. The white squares show dividing cells located both at the periphery and inside the tumorsphere, and appear magnified in the serial sections. **G.** GBM38 sphere. **H1-H3**. The white squares show cells carrying a huge number of lipid inclusions all over the cells. Scale bars represent 10 µm.

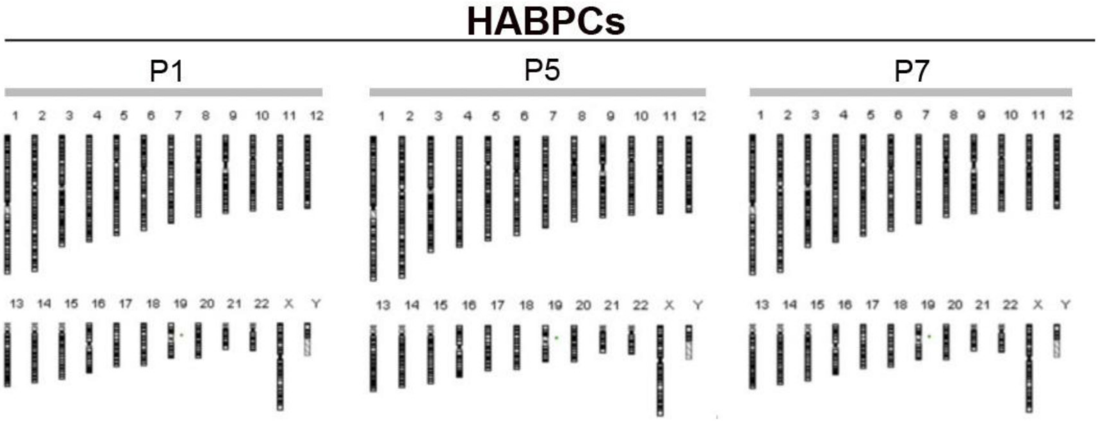


**Supplementary Figure S7: Graphical karyotype representation of Human Adult Brain Progenitor Cells (HABPCs), isolated from adult human brain white matter of patients receiving temporal lobectomy for the treatment of intractable epilepsy.** Comparative Genomic Hybridization was performed on passages 1, 5 and 7. Chromosomal aberrations were absent until the cells became quiescent, which demonstrates the low influence of the isolation procedure and culture conditions on chromosomal instability in non-transformed cells. Keywords: Green line indicates DNA loss.

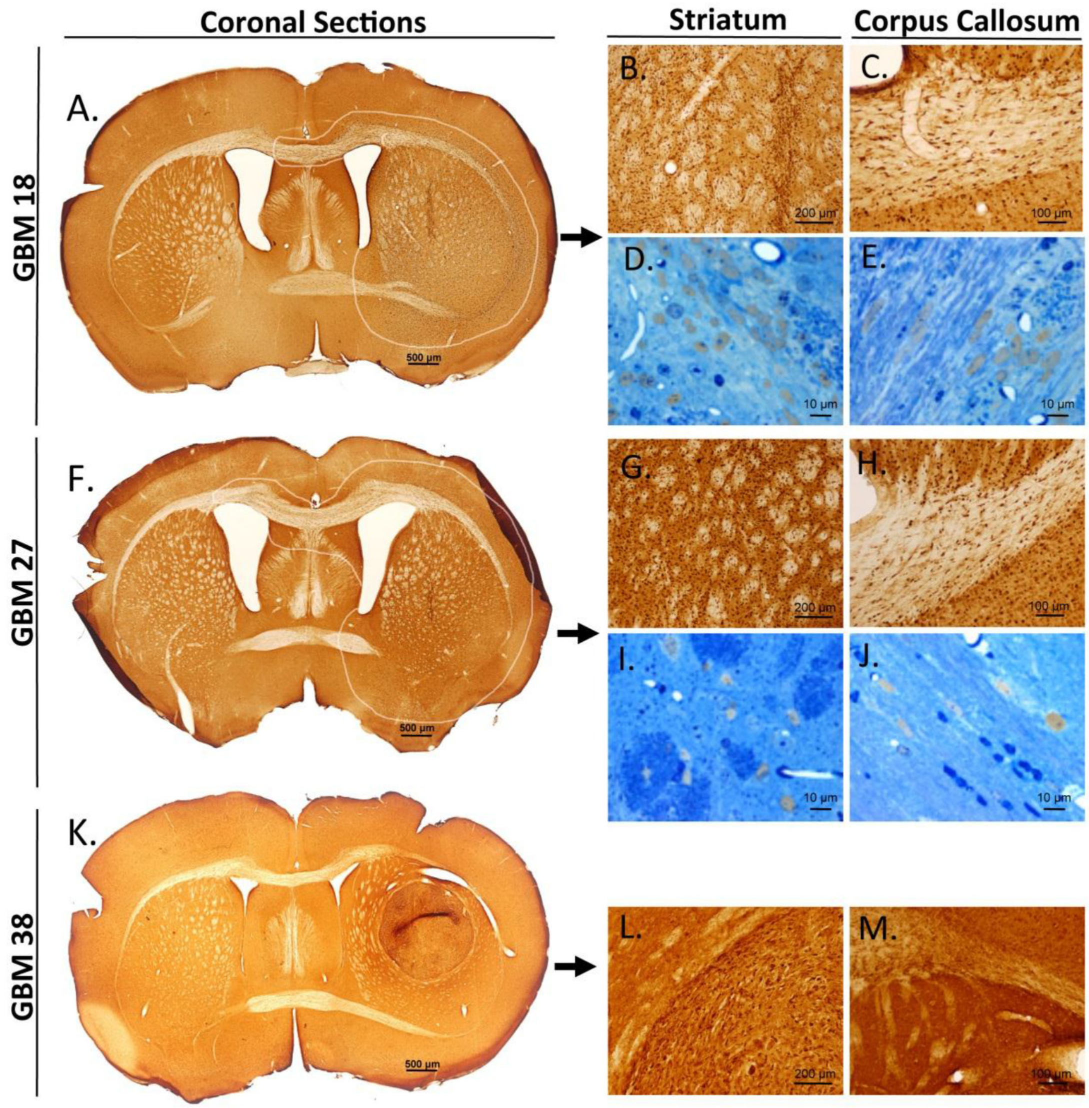


**Supplementary Figure S8: Brain dissemination pattern of CSCs isolated from human samples.** Human cells are revealed by immunostaining with anti-human nuclei. Mice were sacrificed 4 weeks after xenotrasplantation with **A.** GBM18, **F.** GBM27 and **K.** GBM38 without clinical evidences of tumor. Both GBM18 and GBM27 CSC-enriched cultures utilize myelin fibers to disseminate through the parenchymal brain as observed on 50 µm sections stained with DAB **B, C, G** and **H.** and semi-thin sections counterstained with toluidine blue **D, E, I** and **J.** The GBM38 CSC-enriched culture grew as a nodular tumor with well-defined boundaries from very early stages **L, M.**

**Supplementary Table S1: Surgical samples used to derive cancer-stem cells.** Keywords: GBM: Glioblastoma; AA: Anaplastic Astrocitoma; ODG: Oligodendroglioma. Grouped according to *in vitro* growth patterns (*vide supra*).

**See Supplementary File 1**

**Supplementary Table S2: List of primers targeting specific genes designed and validated in our laboratory in the context of previous or present works.** For all cases, cell lines expressing the gene of interest were utilized for primers validation.

**See Supplementary File 2**

**Supplementary Table S3: CSC gene markers used in the present work and their chromosomal location and dotation within the CSC-enriched cultures GBM18, GBM27 and GBM38. Keywords: LOH (loss of heterogeneity).**

**See Supplementary File 3**

**Supplementary Table S4: List of chromosomal alterations in GBM18, by Comparative Genomic Hybridization, along the first 20 passages in culture.** Keywords: white square, deletions; grey square, amplifications; -2, homozygous deletion; -1, heterozygous deletion and +1, heterozygous amplification.

**See Supplementary File 4**

**Supplementary Table S5: List of chromosomal alterations in GBM27, by Comparative Genomic Hybridization, along the first 20 passages in culture.** Keywords: white square, deletions; grey square, amplifications; -2, homozygous deletion; -1, heterozygous deletion; +2, homozygous amplification, +1, hetrerozygous amplification and *, less than 50% of cells.

**See Supplementary File 5**

**Supplementary Table S6: List of chromosomal alterations in GBM38, by Comparative Genomic Hybridization, along the first 20 passages in culture.** Keywords: white square, deletions; grey square, amplifications; -2, homozygous deletion; -1, heterozygous deletion; +2, homozygous amplification, +1, heterozygous amplification and *, less than 50% of cells.

**See Supplementary File 6**

**Supplementary Table S7: Statistically significant DNA sequence gains (19) and losses (20) identified on GBM solid samples by TCGA and analysed in CSC-enriched cultures over the first 20 passages in culture.** Keywords: N, normal; G, gained; L, lost; HL, heterogeneity lost and *, less than 50% of cells.

**See Supplementary File 7**
